# Should cervical favourability play a role in the decision for labour induction in gestational hypertension or mild pre-eclampsia at term? An exploratory analysis of the HYPITAT trial

**DOI:** 10.1111/j.1471-0528.2012.03405.x

**Published:** 2012-06-18

**Authors:** P Tajik, K van der Tuuk, CM Koopmans, H Groen, MG van Pampus, PP van der Berg, JA van der Post, AJ van Loon, CJM de Groot, A Kwee, AJM Huisjes, E van Beek, DNM Papatsonis, KW Bloemenkamp, GA van Unnik, M Porath, RJ Rijnders, RH Stigter, K de Boer, HC Scheepers, AH Zwinderman, PM Bossuyt, BW Mol

**Affiliations:** a Academic Medical CentreAmsterdam; b University Medical Centre GroningenGroningen; c Martini HospitalGroningen; d VU Medical CentreAmsterdam; e University Medical Centre UtrechtUtrecht; f Gelre HospitalApeldoorn; g St Antonius HospitalNieuwegein; h Amphia HospitalBreda; i Leiden University Medical CentreLeiden; j Diaconessen HospitalLeiden; k Maxima Medical CentreVeldhoven; l Jeroen Bosch HospitalDen Bosch; m Deventer HospitalDeventer; n Rijnstate HospitalArnhem; o University Medical CentreNijmegen, the Netherlands

**Keywords:** Bishop score, cervical length, expectant management, gestational hypertension, induction of labour, pre-eclampsia

## Abstract

**Objective:**

To examine whether cervical favourability (measured by cervical length and the Bishop score) should inform obstetricians’ decision regarding labour induction for women with gestational hypertension or mild pre-eclampsia at term.

**Design:**

A post hoc analysis of the Hypertension and Pre-eclampsia Intervention Trial At Term (HYPITAT).

**Setting:**

Obstetric departments of six university and 32 teaching and district hospitals in the Netherlands.

**Population:**

A total of 756 women diagnosed with gestational hypertension or pre-eclampsia between 36 + 0 and 41 + 0 weeks of gestation randomly allocated to induction of labour or expectant management.

**Methods:**

Data were analysed using logistic regression modelling.

**Main outcome measures:**

The occurrence of a high-risk maternal situation defined as either maternal complications or progression to severe disease. Secondary outcomes were caesarean delivery and adverse neonatal outcomes.

**Results:**

The superiority of labour induction in preventing high-risk situations in women with gestational hypertension or mild pre-eclampsia at term varied significantly according to cervical favourability. In women who were managed expectantly, the longer the cervix the higher the risk of developing maternal high-risk situations, whereas in women in whom labour was induced, cervical length was not associated with a higher probability of maternal high-risk situations (test of interaction *P* = 0.03). Similarly, the beneficial effect of labour induction on reducing the caesarean section rate was stronger in women with an unfavourable cervix.

**Conclusion:**

Against widely held opinion, our exploratory analysis showed that women with gestational hypertension or mild pre-eclampsia at term who have an unfavourable cervix benefited more from labour induction than other women.

**Trial registration:**

The trial has been registered in the clinical trial register as ISRCTN08132825.

## Introduction

Gestational hypertension/pre-eclampsia is the most common obstetric complication of pregnancy, with a reported incidence of approximately 10% [1]. Women with gestational hypertension or mild pre-eclampsia constitute about 75% of women with hypertensive disorders of pregnancy at term [2]. For many years the optimal time for delivery of such women has been controversial. Some guidelines recommend labour induction at 37–38 weeks of gestation [3], whereas others endorse expectant management until development of either a maternal or fetal indication for delivery [4]. Those who advise delivery at 37–38 weeks of gestation refer to maternal risks of expectant management, such as progression to severe gestational hypertension, eclampsia or placental abruption, whereas those who recommend expectant management cite the increased rates of caesarean delivery from induction, particularly in those with unfavourable cervix, as well as the increased rates of neonatal morbidities in infants born at 37 + 0 to 38 + 6 weeks of gestation [3].

The HYPITAT trial (Hypertension and Pre-eclampsia Intervention Trial At Term) compared labour induction with expectant management and showed that induction of labour in such women significantly reduces the occurrence of high-risk situations, with a relative risk (RR) of 0.71 (95% CI 0.59–0.86). Moreover, labour induction was not associated with an increase in caesarean delivery (RR 0.75, 95% CI 0.55–1.04) or adverse neonatal outcomes (RR 0.75, 95% CI 0.45–1.26) [5]. As a consequence of this trial, induction of labour has been incorporated into pregnancy-induced hypertension management protocols both in the Netherlands and worldwide [6].

For many years it has been believed by obstetricians that the success of labour induction is determined by the favourability of the cervix and that induction can be selected when the cervix is ripe. In general, there is a reluctance to induce labour in women with an unfavourable cervix because of the fear of increasing caesarean delivery rate [3,7,8] In view of the observed beneficial effect of labour induction observed in the HYPITAT trial, the question is whether cervical ripeness should play a role in the decision to induce labour in these women. In other words, would women with gestational hypertension or mild pre-eclampsia at term and an unfavourable cervix benefit less from labour induction compared with expectant management?

To answer this question, we undertook a post hoc analysis of the HYPITAT trial data, to evaluate the association between favourability of the cervix on admission and the outcomes of labour induction and expectant management.

## Methods

### Study design and participants

**Figure 1:**
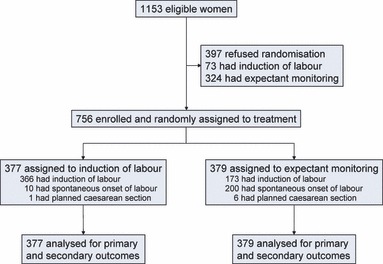
Trial profile.

The background to the trial, methods and baseline characteristics of the randomised women have been previously reported [5]. In brief, the trial included 756 women with a singleton pregnancy and a child in cephalic presentation, gestational age between 36 + 0 and 41 + 0 weeks of gestation, and a pregnancy complicated by gestational hypertension or mild pre-eclampsia. Gestational hypertension was defined as diastolic blood pressure of 95 mmHg or higher measured on two occasions at least 6 hours apart. Mild pre-eclampsia was defined as diastolic blood pressure of 90 mmHg or higher measured on two occasions at least 6 hours apart, combined with proteinuria (two or more occurrences of protein on a dipstick, >300 mg total protein within a 24-hour urine collection, or ratio of protein to creatinine >30 mg/mmol). This study group had been recruited based on a sample size calculation for finding a 6% difference in the risk of developing high-risk situations with 80% power and 5% two-sided type I error rate considering also a 5% protocol violation. Eligible and consenting women were randomly allocated to either labour induction or expectant management (Figure 1).

In the induction group, labour was induced within 48 hours after randomisation. If the cervix was ripe, labour was induced by amniotomy; if labour did not start within 1 hour then augmentation with oxytocin was applied. If the cervix was judged to be unripe, cervical ripening was stimulated using intracervical or intravaginal prostaglandins, according to the local protocol.

In the expectant management group, women were monitored following the local protocol until the onset of spontaneous delivery. Maternal monitoring consisted of frequent blood pressure measurements and laboratory tests. Fetal monitoring consisted of the assessment of fetal movements as reported by the mother, as well as electronic fetal heart rate monitoring and ultrasound examination. Intervention was only recommended in the case of one or more of the following conditions: diastolic blood pressure ≥110 mmHg, systolic blood pressure ≥170 mmHg, proteinuria ≥5 g/24 hours, eclampsia, HELLP syndrome (haemolysis, elevated liver enzymes and low platelet count), suspected fetal distress, prelabour rupture of membranes lasting >48 hours, meconium- stained amniotic fluid or gestational age >41 weeks.

The primary outcome of this trial was a composite measure of high-risk situations, combining the occurrence of maternal mortality, maternal morbidity (eclampsia, HELLP syndrome, pulmonary oedema, thromboembolic disease or placental abruption), progression to severe disease (at least one measurement during antenatal or postpartum period of diastolic blood pressure ≥110 mmHg, systolic blood pressure ≥170 mmHg and proteinuria ≥5 g/24 hours) and major postpartum haemorrhage. Secondary outcomes were caesarean delivery and composite adverse neonatal outcome, consisting of a 5-minute Apgar score <7, umbilical artery pH <7.05, or admission to a neonatal intensive-care unit. Assessment of the cervix before labour had been recorded before randomisation by cervical length measurement (using transvaginal sonography) and Bishop score calculation was by vaginal digital examination.

### Data analysis

There were no missing data for the composite maternal and neonatal outcome measures and the mode of delivery. The Bishop score was missing in 84 women (11% of participants) and cervical length was missing in 48 women (6%). To reduce the risk of biased results and to increase the statistical power of our analysis, we imputed the missing values five times using multiple imputation. We therefore had five complete data sets on which to perform the statistical analyses. All analyses were performed on an intention-to-treat basis and were performed on each complete data set; the results were pooled to obtain the final results.

We developed three separate logistic regression models, for the three outcomes of interest (maternal high-risk situations, caesarean delivery and adverse neonatal outcomes). The predictors in each model were treatment (labour induction versus expectant management), cervical length and an interaction term between treatment and the cervical length. All these analyses were repeated with the Bishop score as the measure of cervical favourability. The fitness of the models was evaluated both graphically and by the Hosmer–Lemeshow test statistic. Data were analysed using R for Windows (Version 2.11.1; R Foundation for Statistical Computing, Vienna, Austria).

## Results

Baseline characteristics of the studied women are summarised in Table 1 and the study outcomes are presented in Table 2. Overall, the HYPITAT trial results indicated that induction of labour reduced the occurrence of high-risk situations without an increase in the risk of caesarean delivery or adverse neonatal outcomes [5]. The median cervical length of the participants was 30 mm (range 0–64 mm) and the median Bishop Score was 3 (range 0–9).

**Table 1:** Baseline characteristics of the participants in HYPITAT trial

Baseline characteristics	Induction of labour (*n* = 377)	Expectant monitoring (*n* = 379)
**Median maternal age in years (IQR)**	29 (26–33)	29 (26–33)
**Median gestational age in weeks (IQR)**	38 (38–39)	39 (38–39)
**Nulliparous**	269 (71%)	272 (72%)
**Diagnosis**		
Gestational hypertension	244 (65%)	252 (67%)
Pre-eclampsia	123 (32%)	123 (32%)
Unknown	10 (3%)	4 (1%)
**Median blood pressure at randomisation in mmHg (IQR)**		
Systolic	140 (140–150)	140 (140–150)
Diastolic	100 (95–100)	100 (95–100)
**Median cervical length in mm (IQR)**	30 (23–37)	30 (22–37)
**Median Bishop score (IQR)**	3 (1–4)	3 (1–4)
**Ethnicity**		
Caucasian	317 (84%)	298 (79%)
Non-Caucasian	35 (9%)	47 (12%)
Unknown	25 (7%)	34 (9%)

IQR, interquartile range.

**Table 2:** The observed outcomes in the participants of the HYPITAT trial

Outcomes	Induction of labour (*n* = 377)	Expectant monitoring (*n* = 379)	*P*-value
**Maternal high-risk situations**	117 (31%)	166 (44%)	<0.0001
Severe systolic hypertension	55 (15%)	88 (23%)	0.003
Severe diastolic hypertension	62 (16%)	103 (27%)	<0.0001
Severe proteinuria	3 (2%)	4 (2%)	0.90
HELLP syndrome	4 (1%)	11 (3%)	0.07
Lung oedema	0	2 (1%)	—
Postpartum haemorrhage	35 (9%)	40 (11%)	0.55
Thromboembolic disease	1 (<1%)	0	—
Placental abruption	0	0	—
**Method of delivery**			
Spontaneous	273 (71%)	253 (67%)	0.091
Vaginal instrumental delivery	50 (13%)	54 (14%)	0.694
Caesarean section	54 (14%)	72 (19%)	0.085
**Adverse neonatal outcome**	24 (6%)	32 (8%)	0.276

**Figure 2:**
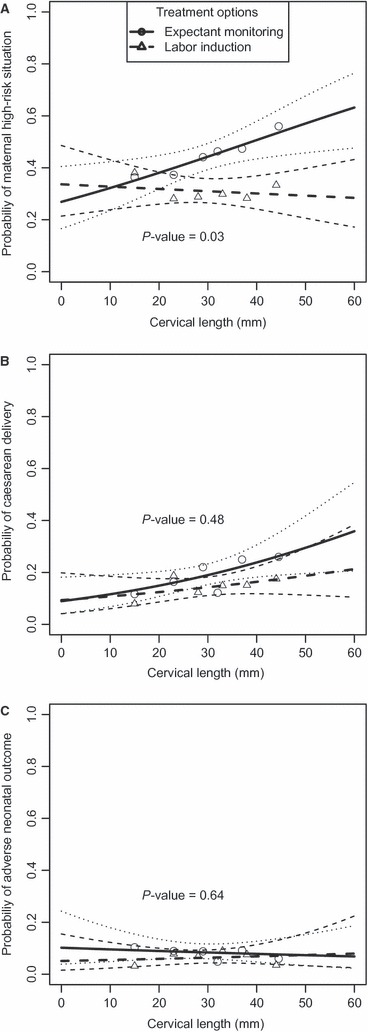
Plots of the primary and secondary outcomes of the HYPITAT trial as a function of the cervical length; (A) risk of maternal high-risk situations, (B) risk of caesarean delivery, (C) risk of developing adverse neonatal outcomes. Lines illustrate model-based estimated risk with 95% confidence intervals (dashed lines). Small circle and triangle markers show the observed risks in the women studied.

Figure 2A shows the probability of developing high-risk situations as a function of cervical length. In women allocated to expectant management, the probability of high-risk situations was higher when the cervix was longer at study entry and each centimetre of increase in cervical length was associated with a 32% increase in the risk of experiencing high-risk situations. In contrast, in women who underwent labour induction the probability of developing high-risk situations was slightly lower when the cervix was shorter; each centimetre increase in baseline cervical length was associated with a 3% risk reduction in experiencing maternal high-risk situations. This difference in association of cervical length to the maternal outcome between the two treatment options was statistically significant (*P*-value of interaction = 0.03; Table 3).

**Table 3:** Modelling of the primary and secondary outcomes of the HYPITAT trial as a function of treatment (expectant monitoring versus labour induction), cervical length and the interaction between treatment and cervical length

Variables	Maternal high-risk situation*		Caesarean delivery*		Adverse neonatal outcome*	
			
	Coeff. ± SE	*P*-value	Coeff. ± SE	*P*-value	Coeff. ± SE	*P*-value
Intercept	−0.71 ± 0.32		−2.19 ± 0.45		−2.99 ± 0.73	
Treatment (EM versus LI)	−0.37 ± 0.50	0.42	−0.06 ± 0.62	0.91	0.72 ± 0.93	0.44
Cervical length (cm)	−0.03± 0.10	0.78	0.13 ± 0.14	0.32	0.09 ± 0.22	0.67
Treatment × cervical length	0.31± 0.14	0.03	0.13 ± 0.19	0.48	−0.14 ± 0.29	0.64

EM, expectant management (treatment coding = 1), LI, labour induction (treatment coding = 0), Coeff., coefficient, SE, standard error.

When woman is managed expectantly (treatment code = 1) every centimetre longer for cervical length at randomisation is associated with a 32% increase in the risk of maternal high-risk situations (exp [−0.03 + 0.31] = 1.32). When the labour is induced (treatment code = 0), every centimetre longer for cervical length at randomisation is associated with a 3% decrease in the risk of maternal high-risk situations (exp [−0.03] = 0.97).

* All models were fit based on the Hosmer–Lemeshow goodness-of-fit test (*P* ≥ 0.75, high *P*-values indicate good fit).

Caesarean delivery was the other important outcome of interest. Among the 377 women who underwent labour induction, 51 (13.5%) developed an indication for caesarean delivery as the result of arrest of first-stage or second-stage of labour, failed instrumental delivery or fetal distress. In the 379 women monitored expectantly, 68 (17.9%) developed an indication for caesarean delivery. As shown in Figure 2B, in both management groups the probability of caesarean delivery was lower when the cervix was more favourable. However, at all levels of cervical length, the risk of caesarean delivery was higher in the expectant management strategy than in the labour induction strategy. For each centimetre increase in the length of cervix at randomisation, the risk of caesarean delivery was higher by 31% if women were managed expectantly and by 14% if they underwent labour induction. This difference in association was not statistically significant (*P*-value of interaction = 0.48).

With regard to the risk of adverse neonatal outcomes, the two groups were comparable. We did not find any significant associations between the cervical length and risk of adverse neonatal outcome (Figure 2C).

The same analyses, repeated with the Bishop score, showed a similar pattern of associations between cervical favourability and the risk of high-risk maternal situation, caesarean delivery and adverse neonatal outcome, but without reaching statistical significance (*P*-value of interaction = 0.23 for high-risk maternal situation, *P* = 0.44 for the risk of caesarean delivery and *P* = 0.95 for adverse neonatal outcome).

We further investigated the increase in the benefit of labour induction in women with an unfavourable cervix, to see if our findings could be explained by the fact that women managed expectantly had a much longer time to develop complications. We divided the studied women into a group with favourable cervix (cervical length < 30 mm) at baseline and a group with an unfavourable cervix (cervical length ≥ 30 mm). Table 4 summarises the results of this analysis. In the induction group, the average time to delivery was 1.9 days in those with a favourable cervix at study entry and 2.6 days when the cervix was unfavourable. In contrast, in women managed expectantly the time from randomisation to delivery was 7.7 days when the cervix was favourable at study entry and 9.1 days when the cervix was unfavourable. This means that women with an unfavourable cervix who underwent induction delivered just 0.7 days later than women who had a favourable cervix, whereas these women delivered on average 1.4 days later if managed expectantly.

**Table 4:** Comparison of the time from randomisation to delivery, the risk of developing maternal high-risk situations, caesarean delivery and adverse neonatal outcomes between women who underwent labour induction or expectant monitoring with or without favourable cervix at randomisation

Management cervix	Induction		Expectant management	
		
	Favourable*	Unfavourable**	Favourable	Unfavourable
Time to delivery (days)	1.91 ± 1.9	2.58 ± 2.8	7.70 ± 5.7	9.11 ± 6.1
Risk of maternal high-risk situation (%)	32.9 ± 47.1	31.7 ± 46.7	38.8 ± 48.9	48.6 ± 50.1
Risk of caesarean delivery (%)	14.6 ± 35.4	14.8 ± 35.6	17.2 ± 37.7	21.1 ± 40.9
Risk of adverse neonatal outcome (%)	5.8 ± 23.8	6.0 ± 25.4	9.4 ± 29.3	8.1 ± 27.4

Values are mean ± SD.

* Favourable indicates cervical length <3 cm.

** Unfavourable indicates cervical length ≥3 cm.

With regard to the outcome, 33% of women with a favourable cervix and 32% of women with an unfavourable cervix in the induction group experienced high-risk situations. This suggests that the risk of developing high-risk situations in the induction group was not affected by cervical favourability. In the expectant management group, an unfavourable cervix was associated with a higher risk of maternal high-risk situations: 39% of women with a favourable cervix experienced maternal high-risk situations compared with 49% of women with an unfavourable cervix. So women with an unfavourable cervix who were managed expectantly had the longest time to delivery and consequently the highest rate of complications.

The time–course of the HELLP syndrome occurrence in the studied women also supports the association between longer time to delivery and disease progression. Overall, 11 cases of HELLP syndrome (3%) were observed in the expectantly managed women and four cases (1%) in women in whom labour was induced. All four HELLP cases in the labour induction group occurred within the first 2 days after randomisation. However, in the expectant management group, 10 HELLP cases developed over a period of 2 weeks after randomisation and one case after 18 days of randomisation.

Table 4 also shows that the risk of caesarean delivery was comparable after labour induction for women with and without a favourable cervix: 14.6 versus 14.8%, respectively. In women managed expectantly, an unfavourable cervix was associated with a higher risk of caesarean delivery: 18.2% with a favourable cervix versus 21.1% when it was unfavourable. We also found no increase in the risk of adverse neonatal outcomes when comparing women with and without a favourable cervix in each treatment group (Table 4).

## Discussion

In the HYPITAT trial, induction of labour was shown to be superior to expectant management in women with pregnancy-induced hypertension or mild pre-eclampsia at term [5]. The key finding of the additional analyses presented here is that the risk of developing high-risk situations depends on the level of cervical ripeness only when women are managed expectantly, in which case a favourable cervix indicates a lower risk of high-risk situations. If labour is induced, the likelihood of high-risk situations is not associated with cervical favourability. As a consequence of this finding, the likelihood of a high-risk condition after expectant management is specifically higher in women with an unripe cervix, which implies that induction of labour is more beneficial to these women. We therefore conclude that labour induction, compared with expectant management, results in a stronger risk reduction for women with gestational hypertension or pre-eclampsia at term who have an unfavourable cervix, with the reduction being most pronounced in women with an unripe cervix.

To our knowledge this study is the first report of the interaction between cervical length and treatment in pregnancy-related hypertension. To replicate this finding in a new data set one would need to have 335 women in each group to be able to find the odds ratio of 1.37 with 80% power at 10% one-sided type I error [9]. The details of this sample size calculation are presented in Appendix S1.

At first glance, this finding may appear to be counterintuitive, if one considers the widely held belief that the decision to induce labour should only be made when the cervix is favourable; because successful induction of labour is related to cervical ripeness [7]. Many studies have unequivocally shown that the rate of labour arrest and caesarean delivery is high in the presence of an unripe cervix [10–12]. This makes obstetricians reluctant to induce labour in women with an unfavourable cervix. In our analyses, we showed a comparable risk of caesarean delivery after labour induction between women with favourable and unfavourable cervices (Table 4). However, as we could analyse the trial data, we had the opportunity to evaluate and compare the parallel association between cervical ripeness and risk of caesarean delivery in comparable women who were managed expectantly. This analysis showed that women with an unripe cervix were at an increased risk of caesarean delivery with expectant management, and that the risk was higher with expectant management than with labour induction.

Nearly 80 years ago, Calkins et al. [13] recognised the importance of cervical assessment in labour induction. For a number of years, preinduction cervical assessment has been accomplished through the use of various measurements and scoring systems, the Bishop score being the one most commonly used. Cervical assessment using the Bishop score was described initially in its application to non-complicated pregnancies in multiparous women [7]. Later, it was shown to predict induction success in nulliparous women as well [12]. There have also been attempts to modify the Bishop score and to create better prediction models for the success of labour induction. Laughon et al. [8], for example, have recently demonstrated that when assessing the cervix, a combination of dilatation, station and effacement was at least as predictive as the Bishop score. A problem, however, is that Laughon et al., just like Bishop and many other researchers, have only looked at the outcome of the induction strategy. Their approach does not assess the effect of cervical status on the outcome of expectant management. As our analysis shows, an unripe cervix is more predictive for an early onset of spontaneous labour in women managed expectantly than for the occurrence of caesarean delivery in women in whom labour is induced. Indeed, the decision for induction of labour is made after a comparison of the consequences of induction versus expectant management, and both should be considered when including cervical ripeness in this clinical decision.

We hypothesised that the main mechanism by which labour induction reduces the risk of a high-risk situation may be through its effect on the time from admission to delivery. We could show that a longer time to delivery is associated with a higher risk of entering into a high-risk situation. This evidence supports the hypothesis that the main reason for better outcomes of labour induction compared with expectant management is that induction shortens the time to delivery and therefore decreases the chance of deterioration of the maternal condition. In both treatment strategies, a shorter cervix was associated with a shorter time to delivery. Women with a long cervix generally deliver later and would be at a higher risk of maternal complications, especially if managed expectantly. Labour induction dramatically shortens the time to delivery and almost invariably results in delivery within 4 days. As a result, women with an unripe cervix, who are at the highest risk when managed expectantly, would have a reduced risk similar to that of other women and hence would obtain the highest benefit.

Evaluation of the occurrence of HELLP syndrome in both groups over time corroborates this hypothesis. We observed that more cases of HELLP syndrome occurred in the expectant management group and most of them occurred within the 2-week period after randomisation. In the women with induced labour all HELLP syndrome cases were observed in the first 2 days, and by terminating the pregnancy using labour induction, the occurrence of more cases of HELLP was prevented.

The HYPITAT study has been criticised because it used a composite outcome measure of high-risk situations, including blood pressures in the high ranges [14]. In this analysis we observed that the endpoint was diagnosed earlier in the induction group than in the expectant management group, which can probably be explained by the fact that women in the induction group are monitored more closely during the first days after randomisation than women in the expectant management group. Nevertheless, despite more monitoring and earlier detection of deterioration in the induction of labour group, more women developed high-risk situations in the expectant management group.

We think that our findings may also hold true for other indications of labour induction in which a progressive disease like pre-eclampsia is present. In such a setting, induction of labour is generally applied because the expected risk of continuation of pregnancy from either a maternal or a fetal perspective is larger than the expected risk of immediate delivery. Consequently, an unripe cervix, which predicts a long time to the onset of spontaneous labour, increases the risk for maternal or neonatal complications. In the case of a ripe cervix, on the other hand, spontaneous labour is likely to start soon, and the risk of such complications is reduced.

## Supporting Information

**Appendix S1.** Formula used for estimation of sample size in new data sets.
