# Supplementary material for: Should cervical favourability play a role in the decision for labour induction in gestational hypertension or mild pre-eclampsia at term? An exploratory analysis of the HYPITAT trial
Source: BJOG. 2012 Jun 18;119(9):1123–30. doi: 10.1111/j.1471-0528.2012.03405.x (PMC3440582; doi:10.1111/j.1471-0528.2012.03405.x)
Supplement: Supplementary file 1 [file bjo0119-1123-SD1.pdf]

Appendix S1

The sample size estimation for a new dataset is done using the following formulae. We put in the formulae the observed 31% event rate of the labour induction group (p_1) and 44% event rate of the expectant management group (p_2). Marker variance (σ^2) is the variance of cervical length (1.157 cm^2). Power was set to 80% and type I error at 10% one-sided for detecting the interaction odds ratio (OR) of 1.37 or larger between the cervical length and treatment. In the formulae below δ is the logarithm of the interaction OR. This results in a sample of size 335 women in each trial arm.

$$n = \frac{(Z_{1-\alpha} + Z_{1-\beta})^2}{\sigma^2 \delta^2} \left(\frac{1}{p_1(1-p_1)} + \frac{1}{p_2(1-p_2)} \right)$$
